# Urinary Extracellular Vesicles: Uncovering the Basis of the Pathological Processes in Kidney-Related Diseases

**DOI:** 10.3390/ijms22126507

**Published:** 2021-06-17

**Authors:** Giulia Cricrì, Linda Bellucci, Giovanni Montini, Federica Collino

**Affiliations:** 1 Laboratory of Translational Research in Paediatric Nephro-Urology, Fondazione Ca’ Granda IRCCS Ospedale Maggiore Policlinico, 20122 Milan, Italy; cricri@ingm.org (G.C.); bellucci@ingm.org (L.B.); 2 Pediatric Nephrology, Dialysis and Transplant Unit, Fondazione Ca’ Granda IRCCS, Policlinico di Milano, 20122 Milan, Italy; 3 Department of Clinical Sciences and Community Health, University of Milano, 20122 Milan, Italy

**Keywords:** extracellular vesicles, kidney disease, cell-to-cell communication, urinary biomarkers, isolation technique

## Abstract

Intercellular communication governs multicellular interactions in complex organisms. A variety of mechanisms exist through which cells can communicate, e.g., cell-cell contact, the release of paracrine/autocrine soluble molecules, or the transfer of extracellular vesicles (EVs). EVs are membrane-surrounded structures released by almost all cell types, acting both nearby and distant from their tissue/organ of origin. In the kidney, EVs are potent intercellular messengers released by all urinary system cells and are involved in cell crosstalk, contributing to physiology and pathogenesis. Moreover, urine is a reservoir of EVs coming from the circulation after crossing the glomerular filtration barrier—or originating in the kidney. Thus, urine represents an alternative source for biomarkers in kidney-related diseases, potentially replacing standard diagnostic techniques, including kidney biopsy. This review will present an overview of EV biogenesis and classification and the leading procedures for isolating EVs from body fluids. Furthermore, their role in intra-nephron communication and their use as a diagnostic tool for precision medicine in kidney-related disorders will be discussed.

## 1. Introduction

The kidneys are the primary filtrating system in the human body. They finely regulate the extracellular fluid volume by altering water excretion and electrolyte concentration, controlling the elimination of waste products, and showing multiple endocrine functions associated with the direct or indirect synthesis of hormones [1].

Kidney diseases, in most cases, are related to multifactorial pathologies such as diabetes and hypertension, as well as immune-mediated conditions. Tests that measure kidney function permit recognition of the damage, monitoring its progression and response to treatment [2]. Kidney biopsy represents a specific diagnostic tool, but it is an invasive and hardily iterative technique, especially in specific patient subgroups, such as children and older people. Recently, urinary peptides [3] and microRNAs (miRNAs) [4] have been suggested as valid alternative markers to classical biochemical measurements [5] of kidney disease progression. Both proteins and miRNAs have been described to be relatively stable in biological fluids, mainly when carried by extracellular vesicles (EVs).

EVs are membrane-surrounded structures released by all cell types and are found in body fluids [6]. EV composition incorporates various bioactive molecules—including membrane receptors, soluble proteins, nucleic acids, and lipids—which can be transferred to target cells [7]. EVs are involved in cell-to-cell communication, influencing recipient cells via direct (i) receptor-binding, thereby transducing a signal, (ii) endocytosis or (iii) fusion with the cell membrane to transfer their molecular contents. EVs may also modify target cells’ microenvironments (i) by interacting with the extracellular matrix (ECM) through integrins or CD44 and (ii) by releasing growth factors/chemokines, thanks to the presence of active enzymes on their surface [8]. EVs have been implicated in cell growth and differentiation, angiogenesis and coagulation, immune modulation, and inflammation [9].

Some of the fundamental qualities of a promising biomarker include good stability, easy accessibility, and the absence of invasiveness during its collection [10]. Based on this approach, EVs could represent an innovative tool for the so-called liquid biopsy, a technique allowing clinicians to screen nonsolid biological tissues such as blood or urine, searching for markers to assess a patient’s condition [11]. Urine contains a large amount of EVs, including those that cross the glomerular filtration barrier (GFB) from circulation, ending up in the urine, and also those originating from the kidney itself. Thus, urine presents itself as an optimal source for biomarker discovery in kidney diseases [12].

This review will present an overview of EV classification and isolation procedures, their role in intra-nephron communication, and their use as a diagnostic tool for precision medicine in kidney-related disorders.

## 2. Extracellular Vesicles 

The term EVs categorizes vesicles based on their size (exosomes, microvesicles, and apoptotic bodies) and release pathways [13]. This review will refer mainly to exosomes and microvesicles, which are the most studied EV populations (Table 1).

Exosomes are small membranous vesicles of endocytic origin ~20–150 nm diameter. In comparison, microvesicles (100–1000 nm in diameter) are large membranous vesicles that are shed directly from the cell plasma membrane and are more heterogeneous in shape and dimension [14].

The release of EVs can occur physiologically, or can be elicited by paracrine factors (cytokines and chemokines) [15,16,17], or physical, chemical, or mechanical stress-stimulated cells [18,19]. EV secretion can also vary in response to external stimuli such as hypoxia [20], inflammation [21,22], and pH variation [23]. Since EVs in individuals with different pathological states are higher than in healthy subjects and show unique signatures [24,25], they have been described as correlating with worsening of the disorders.

Exosomes can generate from intra-luminal vesicles (ILVs) inside multivesicular bodies (MVBs), whereas microvesicles are mainly produced via outward blebbing of the plasma membrane through an asymmetric distribution of cell membrane phospholipids. The complex pathways involved in exosome and microvesicle biogenesis are outside of the scope of this review and have been summarized in the following [26,27,28].

### 2.1. The Molecular Content of EVs

The release of specific EV mediating signals provides a novel mechanism of cell-to-cell communication, allowing cells to transfer their functional cargos to target recipient cells. Accordingly, comprehensive research has been done on the vast repertoire of molecules that can be packaged within EVs, such as proteins, lipids, metabolites, and nucleic acids, as well as DNA reflective of their cellular origin (Table 1) [29].

Proteins associated with the mechanisms responsible for MVB biogenesis, such as tetraspanins (CD63, CD81, CD9), or proteins associated with the endosomal pathway, such as ALG-2-interacting protein X (Alix) and tumor susceptibility gene 101 (TSG101), are commonly found in exosomes. Additionally, cytosolic proteins that participate in membrane fusion events, such as retinoic acid-binding (Rabs) and annexins, are often detected. Moreover, exosomes are heavily enriched in other proteins, including cell-type-specific proteins, such as major histocompatibility complex class II (MHC-II) and CD86, lipid raft components (flotillins) and heat shock proteins (Hsp70 and Hsp90) [29]. Conversely, the protein composition of microvesicles is less well-defined. Proteins selectively recognized as microvesicle-specific include CD40, ADP-ribosylation factor 6 (ARF6), selectins, phosphatidylserine, Rho family members, matrix metalloproteinases, enzymes such as glyceraldehyde 3-phosphate dehydrogenase (GAPDH) and pyruvate kinase, and mitochondria [30,31,32]. Exosomes and microvesicles released in the urine contain proteins derived from integral membrane, such as the adhesion protein CD9 and integrins, and lysosome membrane proteins, such as lysosome membrane protein 2 (LIMP2), lysosome-associated membrane protein 1 (LAMP1) and 2 (LAMP2). Soluble proteins present in the lumen of urinary vesicles comprise α1 antitrypsin, angiotensin-converting enzyme (ACE), tripeptidyl peptidase 1, and the heat shock proteins HSP70 and HSP90, as well as cytoskeletal proteins such as actin, tubulin, and myosin [33,34]. Kidney-specific cells like podocytes can also release EVs that may be identified by the presence of markers for podocyte damage, such as podocalyxin, podoplanin [35], and WT-1 [36,37]. Recent studies revealed that EVs obtained from human urine contain the renal factor Klotho, which is involved in renal homeostasis/physiopathology [38], and whose expression declines with reducing renal function [39].

Among EVs, exosomes represent the fraction most enriched in genetic material [40]. Ratajczak et al. first demonstrated that small vesicles derived from murine embryonic stem cells (ESCs) contained mRNAs, coding for several pluripotent transcriptional factors. These vesicles had functional activity on target cells [41]. Furthermore, the research conducted by Valadi and colleagues [42] demonstrated that mRNAs transferred through the exosomes were biologically active since they were delivered to the target cells, resulting in protein translation. Depth analysis of EV content revealed that exosomes contained other species of small non-coding RNAs (ncRNAs) such as miRNAs and long non-coding RNAs (lncRNAs).

Urinary EV’s transcriptome is mainly composed of RNAs of kidney origin. One case is the mRNA encoding CD2-associated protein (CD2AP), which was detected in urinary EVs coming from healthy individuals and glomerular disease patients [43]. As numerous high abundant miRNA species have been associated with various kidney diseases, urinary EV miRNA signature has been proposed as a valuable non-invasive source of information of events occurring in the kidney [44]. Two miRNAs (miR-29c and miR-26a) have been identified in urinary EVs and correlated with progressive decrease in glomerular filtration in kidney pathologies [45,46].

EVs can also be active carriers of different types of DNA [47], including single-stranded DNA (ssDNA), double-stranded DNA (dsDNA), and mitochondrial DNA (mtDNA). However, the relative abundance of different DNA cargo in EVs and their biological function are still unclear.

### 2.2. Methods of EV Isolation

**Figure 1:**
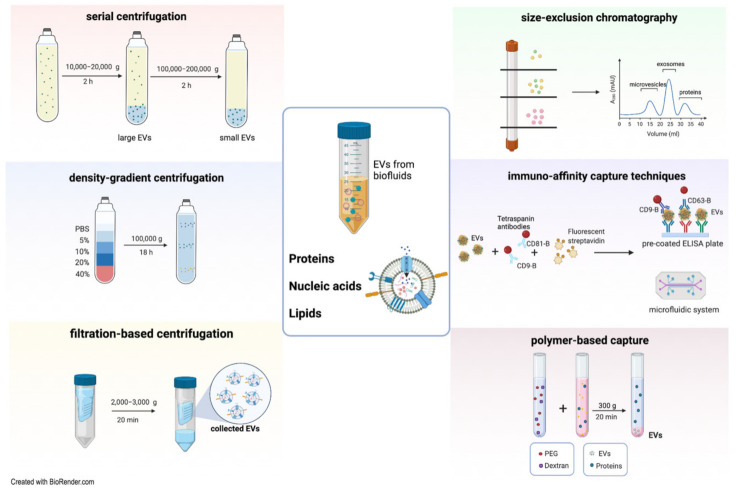
Overview of the most common techniques used for EV isolation from body fluids.

The use of EVs as a biomarker requires developing isolation procedures to collect EVs from complex matrices and small volumes of fluid and to eliminate unwanted non-EV contaminants [48]. One of the main limitations in the study of EVs is the current lack of a standardized method for isolation. Therefore, different isolation protocols have been developed (Figure 1). However, the choice of an adequate technique for the isolation of EVs is critical because of the complex biologic source from which EVs are separated and the specific downstream application/study [49]. The most commonly used EV purification methods include serial ultracentrifugation, density-gradient centrifugation, precipitation, filtration, size exclusion chromatography, and immunoprecipitation/affinity capture [13]. Interestingly, they may be employed either individually or in combination.

Ultracentrifugation, generally used for EV isolation from body fluids and cell culture media, is a conventional technique characterized by a high recovery of specific enrichment of EV subtypes. This method is the most frequently used approach to isolate urinary EVs. [34,50]. The general protocol consists of multiple sequential ultracentrifugation steps allowing vesicles to sediment based on their sizes. The low speed (10,000–20,000× *g*) will enrich the preparation of large vesicles as microvesicles, and the high speed (100,000–200,000× *g*) will be effective in concentrating an exosome-enriched EV population [51]. Apart from being a time-consuming procedure, ultracentrifugation may lead to unreliable results, since the separation of different EV subtypes could be contaminated with non-vesicular elements wrongly interpreted as integral EV components in downstream analyses. Indeed, this isolation methodology appears to be less efficient at purifying EVs from the urine of nephrotic syndrome (NS) patients, since the highly abundant soluble proteins collected in the pellet [52] may complicate the detection of selective EV proteins.

An additional step with density-gradient centrifugation—using sucrose or iodixanol gradient solutions—is commonly required to enhance the purity of EVs. This different approach separates EVs according to their flotation densities, together with their different sizes [53]. Top-to-bottom or bottom-to-top loading procedures can be applied to separate particles, along with the different densities of the solution [54], including different types of urinary EVs [55]. Like differential ultracentrifugation, this procedure is time-consuming. Additionally, it employs special requirements, and the extreme g-forces used for EV purification may lead to their disruption and loss of their biological activity [56].

Filtration-based methods can also isolate specific subsets of EVs. Sequential filtration enables the separation and collection of differently sized EV populations by pressure or centrifugation through the use of pore size-containing membrane filters with different exclusion limits. The efficiency of the filtration-based method to purify EVs depends on several prior centrifugation steps to discard cells and larger vesicles or apoptotic bodies, thus enabling specific capture of distinct EV subsets. To collect urinary EVs, it is possible to use the nanomembrane ultrafiltration approach, which has a lower limit of urine sample volume and is faster and simpler [57]. Unfortunately, this common size-based separation technique cannot avoid losing EV yield due to clogging and trapping in the filter unit [58]. Although filtration is less time-consuming and does not involve special requirements, the applied shear force may induce particle deformation [59].

The immunoaffinity capture technique is based on antibodies to specifically recognize antigens according to their expression on the surface of EVs. Antibodies specific for surface proteins of EVs may be coupled to magnetic beads, microfluidic technologies, other material supports (pre-coated ELISA plate), and resins [60]. This procedure enables direct capturing of all EVs or selective subpopulations of EVs, guaranteeing increased purification efficiency. Due to the lower vesicular yield reached with immunocapture, this procedure is often used in conjunction with other techniques, such as ultracentrifugation and filtration [61]. Recently, a high-resolution fluorescence analysis and cytometric sorting technology was developed to improve isolation of tumor and immune-derived EVs from cell cultures using antibodies-based detection [62]. This system is a powerful method for single EV phenotyping, permitting study of different subpopulations of particles overcoming their heterogeneity.

New isolation methods of EVs rely on organic solvents or specific polymers, such as polyethylene glycol (PEG), which typically decrease vesicular solubility and cause precipitation through low-speed centrifugation [63]. Polymer-based approaches are easily performed, but collected EVs are usually impure and an added step of protein removal is required to eliminate coprecipitated proteins [48]. Additionally, size exclusion chromatography, also known as gel filtration, accurately separates EVs based on their molecular size as they pass through a column packed with a stationary phase consisting of heterogenous polymeric beads with diverse pore sizes [64]. Size exclusion chromatography (SEC) can be paired with other techniques to achieve a relevant purity and yield [65,66]. Despite SEC overtaking other techniques based on the purity of the isolated EVs, that works to the detriment of their total yield, making it difficult to scale SEC up for clinical purposes [54]. The different EV isolation methods show possible benefits/weaknesses and, more importantly, distinct specificity in purity and variances in the molecular content of the isolated vesicles [67,68,69]. For this reason, the Minimal Information for Studies of EVs 2018 (MISEV2018) guidelines proposed the generation of standardized methodologies to be combined for the purification of EVs from biofluids in order to avoid the generation of misleading results [13].

## 3. EVs in the Kidney: Mechanism of Cell-to-Cell Communication

**Figure 2:**
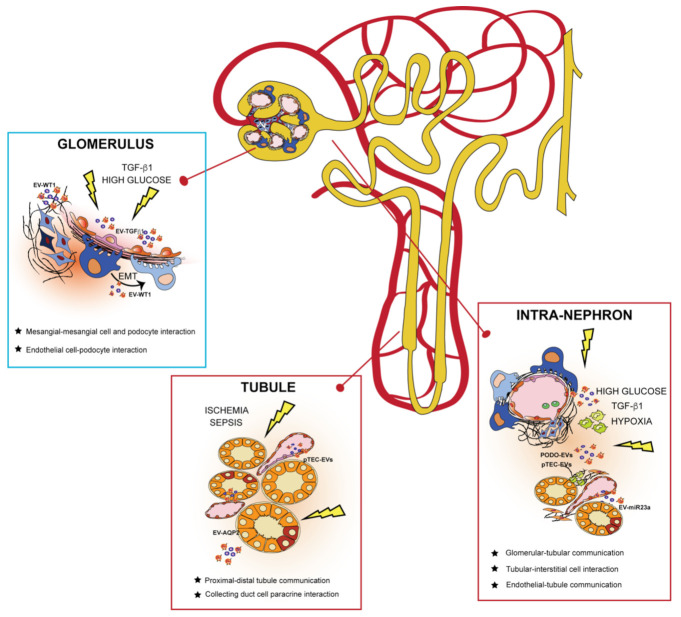
Extracellular vesicles (EVs) govern intracellular communication between intra-nephron compartments. EVs released from different glomerular cells and tubule segments can interact in the same physical compartment or mediate communication from distant nephron sections in physiological and pathological conditions. In the glomerulus, EVs released from mesangial and endothelial cells (such as EV-TGFβ) mediate a paracrine response to injury by affecting neighboring kidney cells. EVs of podocyte origin (EV-WT1) are released in the urine during pathological conditions, behaving as biomarkers of glomerular kidney damage. In the tubule, EVs are secreted by tubular epithelial cells (TECs) to maintain fluid homeostasis and electrolyte balance (EV-AQP2) in the body. After an injury, EVs from both podocytes (PODO-EVs) and proximal TECs (pTEC-EVs) mediate intra-nephron interaction with interstitial fibroblasts and tissue-infiltrating macrophages, promoting progression to chronic kidney disease.

EVs are potent intercellular messengers carrying different molecular cargos implicated in communicating signals throughout the nephron (Figure 2) [70]. In this context, all the cells composing the urinary system can release EVs involved in cell crosstalk, contributing to kidney physiology and pathogenesis [71].

### 3.1. EVs and Glomerulus

The function of the glomerulus is to filter the blood through the glomerular basal membrane (GBM) and visceral epithelial cells called podocytes [72]. EVs are constitutively shed by different glomerular cells in physiological conditions, and their release is facilitated by abnormal stimuli associated with diseases [73]. EVs determine the physiological transfer of molecules, such as miRNAs, between glomerular endothelial cells (GEC) and podocytes. In fact, it has been shown that podocyte cytoskeletal stability and VEGF secretion largely depend on the integrity of this communication [74]. Moreover, proteomic studies of podocyte-derived EVs in urine have demonstrated the presence of enzymes involved in sodium and calcium homeostasis, such as catechol o-methyltransferase (COMT) and family with sequence similarity 26, member E (FAM26E) [75].

Under high glucose conditions, mimicking diabetic nephropathy (DN), glomerular mesangial cells can produce exosomes enriched with renin and angiotensinogen. These exosomes, in turn, can increase the renin-angiotensin system components in target cells and stimulate fibronectin synthesis, thus impairing glomerular functionality [76]. In another experimental study, it was demonstrated that glomerular endothelial cells (GEC), after exposure to high glucose concentration, released EVs enriched with transforming growth factor (TGF) β1 mRNA. EVs enriched with TGFβ1 can regulate the crosstalk between GEC and podocytes, mediating epithelial-mesenchymal transition (EMT) and damage in podocytes [77]. Podocytes are the main targets of many glomerular diseases; thus, podocyte-derived EVs may represent a potential mechanism leading to a decline of kidney function in these disorders [78].

### 3.2. EVs and Kidney Tubules

Kidney tubules are involved in several regulatory functions in normal physiology and the pathogenesis of kidney diseases [79]. Importantly, in this part of the kidney, most of the small solutes filtered in the glomerulus are reabsorbed. Tubular epithelial cells (TEC) can secrete EVs responsible for modulating the expression of solute-transporting proteins in other neighboring cells [80]. In this regard, proximal TEC (pTEC)-derived EVs mediate the downregulation of physiological epithelial sodium channel activity in the collecting duct cells, thereby regulating the appropriate sodium and water retention [80]. Another role of pTEC-derived EVs in kidney physiology is to reduce intracellular reactive oxygen species (ROS) in both distal tubules and collecting duct cells during their uptake, thus contributing to tubular homeostasis [81].

Moreover, collecting duct cells, cultured in the presence of desmopressin, release and absorb higher levels of functional aquaporin-2 (AQP2)-enriched EVs, which in turn act on other collecting duct cells, increasing water permeability [82]. Notably, specific miRNA cargo may also prompt urinary EVs (uEVs) to modify the expression of local transporters and channels as detected by next-generation sequencing analysis [83]. In particular, uEVs impair kidney outer medullary potassium channel (ROMK) and plasma membrane Ca^2+^ ATPase (PMCA1) protein expression in collecting duct cells, thus suggesting a regulatory role for EVs in calcium and potassium active reabsorption [83].

EVs derived from injured TEC may potentially reflect the onset of tubular damage. After exposure to an injury, increases in tubule-derived kidney solute and water transporters such as AQP2, activating transcriptional factor 3 (ATF3), and fetuin-A [36,82,84] have been reported. Notably, the levels of these transporters found within tubule-derived EVs are consistent with those observed in their respective cells. Moreover, a prolonged exposure to glucose, leading to DN development, changes naïve renal pTEC by activating specific signaling pathways (markers of TGFβ, EMT, and endoplasmic reticulum stress) mediated by renal TEC-derived EVs [85].

### 3.3. EVs in Communication from Different Nephron Sections

Within the kidney, EVs may be involved in conveying messages to different sections of the nephron. Concerning glomerulus-tubule signaling, it is likely that microvesicles released from podocytes mediate the transition from glomerular injury to tubular fibrosis. Indeed, vesicles can interact with pTEC, inducing the activation of p38 mitogen-activated protein kinases (MAPK) and CD36 to accelerate a profibrotic process [86]. Emerging evidence suggests that EVs may actively take part in the fibrotic process [87]. In particular, TGFβ1-enriched exosomes secreted by ischemic damaged TEC can be transferred to normal kidney cells, which might in turn activate fibroblast proliferation and the development of kidney fibrosis [22].

Hypoxia-inducible factor-1 (HIF1-α) can also influence TEC-derived EV cargo by promoting miR-23a enrichment. After entering intra-renal myeloid cells, miR-23a leads to tubulointerstitial inflammation by promoting the classical M1 pro-inflammatory phenotype of tissue macrophages [88]. Upon injury signals, podocytes can also secrete specific EV-enclosed miRNAs, thereby activating the p38 signaling pathway in TEC, leading to their damage and contributing to the development of kidney fibrosis [89]. Interestingly, endothelial cells (EC) present in the urinary tract appear to mediate the production of HIF-1α/vascular endothelial growth factor (VEGF)-A from proximal TEC via microvesicle-mediated crosstalk, thus influencing kidney disease outcomes [90].

### 3.4. Non-Kidney Cell EVs in Intra-Renal Communication

Different cells such as fibroblasts and immune cells are essential elements of intra-renal crosstalk, a phenomenon that is also frequently associated with EV secretion [91]. In their study, Eyre et al. demonstrated that monocytic-derived EVs contributed to glomerular inflammation by inducing podocytes to produce high levels of pro-inflammatory molecules, such as Monocyte Chemoattractant Protein-1 (MCP-1) and Interleukin (IL)-6. Moreover, the release of monocytic-derived EVs activated podocytes to induce VEGF production, thus constituting a critical mechanism of glomerular permeability [92]. Additionally, excessive accumulation of calcium oxalate crystals in the interstitium is a leading cause of progressive inflammation in kidney stone disease. Macrophages are the effector cells immediately recruited to the inflammatory site and implicated in eliminating deposited crystals. However, at later time points, they exhibit pro-inflammatory effects by increasing exosome-induced IL-8 production from renal TEC, which subsequently enhances neutrophil migration into the interstitium, thus worsening the inflammatory process [93].

## 4. uEVs as Biomarkers for Personalized Medicine

In clinical practice, the most commonly used markers of kidney disease, including serum creatinine, estimated glomerular filtration rate (eGFR), blood urea and proteinuria, and albuminuria, are insufficient. The main limitations are the inability to reflect early functional changes in the kidney and the lack of prediction of chronic kidney disease occurrence [94]. Therefore, identifying novel biomarkers, whether used alone or in combination with conventional ones, can overcome these limitations, facilitate better definition of kidney pathophysiology and make personalized therapeutic treatments possible. In this context, urine contains a large amount of EVs, both in physiological and pathological conditions, which can reflect alterations in specific cellular compartments [95]. Moreover, because of the glomerular architecture, large circulating EVs in physiological conditions cannot cross the GFB. Therefore, uEVs mainly originate from epithelial and parenchymal cells facing the urinary space [22]. For this reason, they have been proposed as promising, non-invasive liquid biopsies for a variety of kidney diseases [96]. Moreover, the optimization of protocols for the purification of exosomal RNA has allowed for their use as a novel class of easy-to-obtain sources of genetic biomarkers [65].

## 5. uEVs as a Liquid Biopsy in Primary Kidney Diseases

The specific composition of uVEs has been directly correlated to some primary kidney diseases. The use of uEVs could represent both an early diagnostic approach and a prognostic tool capable of facilitating individual therapeutic decisions [97]. In Table 2, we reported published papers in which uEVs were applied for the clinical diagnosis of kidney-related diseases.

### 5.1. Nephrotic Syndrome

NS is the most common glomerular disorder in children, whose clinical picture is characterized by edema, proteinuria, and hypoalbuminemia. Ninety percent of pediatric patients respond to steroids (steroid-sensitive), while 10% are steroid-resistant [112]. Urine from patients with NS contains large amounts of molecules, so it is currently a critical biological matrix for valid biomarkers in this disease. The podocyte-specific zinc finger transcription factor Wilms’ tumor 1 (WT1) was found in the urine of NS patients [37]. A well-defined correlation of WT1 expression with the development of NS in a cohort of 71 patients was described. However, no significant differences in reactivity to steroids emerged in that study [37].

Conversely, a noticeable decrease of WT1 expression was found in urinary EVs of patients with NS in remission or six steroid-sensitive subjects [36]. Subgroup differences were also highlighted by Santorelli et al. [113], who tried to build a statistical model capable of classifying NS subgroups based on the patient’s uEV profile. The authors demonstrated that uEVs from steroid-resistant patients presented a peculiar protein pattern, different from the other idiopathic NS patients. Furthermore, these protein profiles were determined to be specific for idiopathic NS since they were different from the uEV protein patterns of other diseases (hereditary tubulopathies) and healthy controls [113]. Children with NS and a histological picture of focal segmental glomerulosclerosis showed a different composition of urinary exosomes—e.g., in miRNA content (higher levels of miR-193)—than children with minimal change disease [98]. In recent research, the miRNA profile of uEVs from 129 NS children was analyzed by high-throughput sequencing analysis, followed by a quantitative reverse transcription-polymerase chain reaction verification. Five altered miRNAs were identified according to disease progression and treatment. The results suggested that miR-194-5p and miR-23b-3p were correlated with urine protein content and could reflect the severity of the disease [99].

### 5.2. Nephritic Syndrome

Nephritic syndrome is an inflammation of the glomerulus, leading to hematuria, reduced kidney function, hypertension, and edema.

Glomerulonephritis (GN) is the primary expression of this involvement, which can occur in the context of systemic (lupus nephritis and vasculitis) or isolated (post-infectious and IgA GN) diseases. Kidney biopsy is often the definitive diagnostic test. A pioneering study performed a protein profiling of urinary exosomes of patients with IgA GN and thin basement membrane nephropathy, compared to healthy volunteers [100]. Among the 1877 proteins identified, four proteins (aminopeptidase N, vasorin precursor, a-1-antitrypsin, and ceruloplasmin) were selected as biomarkers to distinguish between the 2 GN differentially expressed [100]. It was subsequently confirmed that urinary excretion of EVs correlated with the extent of histological damage (mesangial hypercellularity, crescents, and endocapillary hypercellularity). Furthermore, by investigating the EV content, it emerged that chemokine ligand 2 (CCL2) (motif CC) mRNA, linked to tubulointerstitial inflammation and C3 deposition, was specifically expressed in uEVs of patients with GN, compared to healthy controls [101]. EVs released by kidney cells were proposed to mediate multiple pathological processes in lupus nephritis [114]. For instance, EV-containing immune complexes have been described in the urine of Systemic Lupus Erythematosus (SLE) patients [115].

The use of EVs as possible biomarkers for the diagnosis or therapeutic monitoring of LN has been deeply investigated. The miR-146a—carried by uEVs—has been proposed as a biomarker of kidney damage [116]. It was able to differentiate patients with active lupus nephritis compared to the control group and lupus patients without nephritis [116]. In 2020, the same group showed—in 41 lupus nephritis patients—that uEV-miR-146a levels inversely correlated with lupus activity, proteinuria, and histological features [102].

In a study including 32 patients with biopsy-proven lupus nephritis, miR-29c was predictive of chronic histological damage and glomerulosclerosis but not glomerular function decrease [103]. Finally, a study on the long-term follow-up of SLE patients (*n* = 41) with the active or inactive disease showed that two miRNAs, let-7a and miR-21, were significantly downregulated in patients with active LN, compared to the inactive ones, suggesting their use as therapeutic biomarkers [104].

### 5.3. Ciliopathies

Ciliopathies are genetic disorders characterized by defects in ciliary-related proteins, causing a variety of pathological manifestations, including polycystic kidney diseases (PKD) and nephronophthisis [117]. Depletion of a protein component of the Exocyst complex, which is involved in cilia formation, correlated with the regulation of EV biogenesis/release and cilia development in kidney tubular cells, both *in vivo* and *in vitro* [118]. In a cohort of 12 children with nephronophthisis-related ciliopathy and age- and gender-matched controls, 156 proteins were identified as differentially expressed in the uEVs of the two groups [105]. Five of the most distinct proteins in the uEVs from nephronophthisis patients were correlated with chronic kidney disease, supporting EVs’ role as a biomarker of kidney damage progression in this pathology [105].

Among the commonly inherited kidney diseases and ciliopathies, there is PKD, caused by mutations in genes encoding for proteins that regulate the normal function of primary cilia including polycystin (PC) 1, PC2, and fibrocystin [119]. Studies on human autosomal recessive PKD patients identified an abnormal accumulation of EVs along cilia in the kidneys [106], confirming a correlation between ciliopathies and EVs [120]. Moreover, the PKD1- and PKD2-encoding proteins PC1 and PC2 are present in ciliary derived-EVs and urinary exosome-like vesicles and likely play a relevant role in maintaining vesicular architecture [71,121]. Urine-derived exosomes of PKD patients also contain other proteins such as cystin and ADP ribosylation factor-like 6 [71]. The lectin profile of uEVs can be another valid method to monitor PKD progression, since different lectin patterns were identified between PKD patients and healthy controls [122]. This suggests possible disease-specific modifications in the molecular content carried by EVs. Data from Ward’s group showed that transmembrane protein-2 (TMEM2) was consistently more abundant in uEVs derived from PKD1 patients than in controls [121], showing an inverse relationship between PC to TMEM ratio and kidney volume. Therefore, urine exosomal PC1 to TMEM2 or PC2 to TMEM2 ratio could provide a functional diagnostic parameter to monitor PKD [121].

### 5.4. Tubulopathies

Tubulopathies, such as Gitelman syndrome (GS) and Bartter syndrome (BS), are rare genetic salt-losing disorders caused by loss-of-function mutations in genes linked to renal tubular electrolyte transport. Modification in electrolyte balance caused extracellular volume contraction and increased activity of the renin-angiotensin-aldosterone axis [123,124]. Interestingly, patients with the disturbed tubular sodium reabsorption system secreted uEVs depleted of the typical solute-coupled transporters necessary to maintain normal sodium and chloride excretion in the kidney [95]. Another type of transport protein disorder affecting urinary concentration includes diabetes insipidus, caused by an impaired water permeability of the collecting ducts due to the inability of the kidneys to respond to arginine vasopressin (AVP) stimulation [125]. The impaired responsiveness to AVP stimulation has been correlated with a loss-of-function mutation in the vasopressin V2 receptor gene *AVPR2* in patients with lower levels of AQP2 protein in urine [126].

Interestingly, exosomes released by kidney collecting duct cells, treated with the synthetic vasopressin analogue desmopressin, stimulated both AQP2 expression and water transport in untreated cells. This event was accompanied by a change in exosomal protein composition that also occurred *in vivo*, where an increase in the AQP2: flotillin-1 ratio in uEVs was detected following desmopressin treatment in rats [82]. As such, by measuring the AQP2 protein abundance in urine-derived exosomes, it will be possible to detect alterations in vasopressin-controlled epithelial events, which may serve to characterize the phenotype of rare inherited transport protein disorders.

## 6. uEVs as a Liquid Biopsy in Secondary Kidney Diseases

In addition to primary kidney pathologies, there are a series of diseases that secondarily affect the kidney and progress to chronic kidney disease, demanding new noninvasive biomarkers of disease progression.

### 6.1. Diabetic Nephropathy

Evidence of kidney damage has been detected in about 30–40% of patients with type 1 or type 2 diabetes (T1D and T2D, respectively) [127]. The first hallmark of DN is the development of glomerular damage with podocyte death and proteinuria development [122]. Urine composition has been investigated, searching for more specific diagnostic criteria in DN. Urinary WT1 protein, shed by kidney epithelial cells and incorporated into uEVs, was proposed as a biomarker of DN. A study involving 48 patients with T1D found that WT1 expression in urinary exosomes was significantly higher than in controls [107]. The WT1 levels were associated with a significant increase in urine protein-to-creatinine ratio, albumin-to-creatinine ratio, and serum creatinine, as well as a decline in eGFR [107]. In a study aimed to understand the function of uEV-miRNAs in children with T1D, urine samples were collected from 30 healthy controls and 30 T1D patients, resulting in the identification of 2 miRNAs, miR-424 and miR-218, which could be predictive of the patient’s prognosis [108]. Another study applied genome-wide miRNA profiling of uEVs in T1D patients [109]. The authors identified a cluster of differentially expressed miRNAs correlated with hemoglobin A1c (HbA1c) levels in T1D patients and associated with fibrosis-related pathways [109].

### 6.2. Obstructive Nephropathy

Congenital Obstructive Nephropathy can be caused by the presence of posterior urethral valves (PUV) [128] and pelvic ureteric junction obstruction [129], which can lead to chronic kidney damage.

In a case-control study, specific biomarkers of obstructive nephropathy were measured in whole urine and uEVs [110]. In children with PUV, urinary excretion of exosomal AQP1 was significantly decreased, whereas those of TGFβ1 and the L1 cell adhesion molecule (L1CAM) were significantly increased. Moreover, TGFβ1 expression inversely correlated with eGFR in these patients [110]. iTRAQ quantitative proteomic profiles of amniotic fluid-derived exosomes from women at 15–25 weeks of gestation who were diagnosed for congenital ureteropelvic junction obstruction (UPJO) showed significant alterations in their protein contents [111]. In particular, angiotensin-converting enzyme (ACE) and aminopeptidase N (AP-N) were significantly decreased in the amniotic fluid exosomes of women with UPJO. These proteins correlate with suppressed cell proliferation, elevated ROS production, and increased pro-inflammatory cytokine levels in human kidney (HK)2 cells [111] and support their use as prognostic factors of kidney damage occurrence in urinary tract obstruction disorders.

## 7. EVs in Clinics: Perspectives and Limitations

In the past years, human-derived naïve and engineered exosomes have been applied in clinical trials as therapeutics for cancer and genetic diseases [130]. In the context of biomarker discovery, the scientific community has shown a growing interest in developing standardized methods for EV studies.

For EV diagnostic applications, the established assays should be sensitive, rapid and capable of specifically detecting the biomolecules present in the vesicles [131]. Although uEVs represent only 3% [132] of the entire urinary proteome, a growing interest in their use as diagnostic and prognostic biomarkers has been highlighted. The translational use of EVs implies their clinical specificity. Although numerous molecular cargos have been correlated with defined pathological states such as cancer, their specificity cannot be assured [24]. Indeed, the same molecules can be activated in multiple pathological conditions and released in EVs. These molecules are usually involved in common cellular processes, such as proliferation, cell death, migration, and so on [24]. Clinical specificity can be improved by the generation of complex panels of EV-associated biomarkers in which EV number, phenotype, and molecular content are combined and correlated to specific diseases.

Although heterogeneity of EVs can be another limit in their use as biomarkers, their ability to reflect cellular complexity can allow monitoring of a pathological state in all aspects of its progression [133]. At the same time, a growing number of technologies granting the analysis of specific EV subpopulations have been developed [134,135]. These new techniques focus on the improvement of EV yield and purity, trying to eliminate contaminants accounting for the modification of the chemical/biological composition of EVs, as well as their physical properties [136].

## 8. Conclusions

EVs are active biological agents that play an essential role in intercellular communication, showing beneficial or detrimental effects on recipient cells based on their origin. The potential of EVs to mirror changes in different compartments of the nephron and their release in the urine emphasizes their role in the pathophysiology of kidney-related diseases. Moreover, uEVs provide a protected microenvironment for molecular content that can be easily screened, searching for stable biomarkers for personalized therapies. Despite the observation that vesicular biomarkers can be more reliable and better correlated with clinical parameters than non-vesicular biomarkers, a critical evaluation of the use of uEVs in kidney-related diseases should be considered. Variability of the isolation methods, the ambiguity of EV classification, inexperience regarding the best EV sources and the existence of misleading factors affecting EV content independently from the kidney pathology are many of the elements that should be considered for future study and application of uEVs in clinical settings.

## Figures and Tables

**Table 1:** Overview of EVs: their size, origin, composition and isolation techniques.

	Exosomes	Microvesicles
**Size (nm)**	20–150	100–1000
**Origin**	MVB fusion with plasma membrane	Outward blebbing of plasma membrane
**Release process**	Constitutive/Cellular Activation	Constitutive/Cellular Activation
**Pathways**	Tetraspanins-dependent	Cytoskeleton reorganization- and Ca^2+^-dependent
**Composition**		
Proteins	CD9, CD63, CD81, Alix, TSG101, Rabs, annexins, MHC-II, CD86, signaling-, oncogenic-, integrin-, adhesion molecules, flotillins, Hsp70 and Hsp90	Annexins, flotillins, Alix, TSG101, CD40, ARF-6, selectins, phosphatidylserine, Rho family members, MMP2, ADAM, GAPDH, pyruvate kinase, proteins localized to centrosome, nucleolus, cytoplasm and mitochondria
Nucleic Acids	mRNAs, miRNAs, lncRNAs, mtDNA, gDNA, nDNA, dsDNA	mRNAs, miRNAs, gDNA
**Isolation Method**	UC, DG-UC, filtration, immune- affinity, PEG precipitation, SEC	UC, DG-UC, filtration, SEC

MVB: Multivesicular bodies; TSG101: tumor susceptibility gene 101; HSP: heat shock protein; ARF-6: ADP ribosylation factor 6; MMP: matrix metalloproteinase; ADAM: a disintegrin and metalloproteinase; GAPDH: glyceraldehyde 3-phosphate dehydrogenase; mRNAs: messenger RNAs; miRNAs: microRNAs; lncRNAs: long non-coding RNAs; sRNAs: small RNAs; mtDNA: mitochondrial DNA; gDNA: genomic DNA; nDNA: nuclear DNA; dsDNA: double stranded-DNA; UC: ultracentrifugation; DG-UC: density gradient-ultracentrifugation; SEC: size-exclusion chromatography.

**Table 2:** uEV biomarkers for clinical diagnosis of kidney-related diseases: a summary of human studies.

Disease	No. of Patient	Target	uEV Isolation	Detection Method	Main Results	Ref.
	25 patients and 5 HD	WT1	Differential UC	WB	-WT1 was increased FSGS patients compared with healthy volunteers or SSNS patients. -Urinary exosomal WT1 was decreased in patients in remission for either FSGS or SSNS or following steroid treatment in six SSNS subjects.	H. Zhou, 2013 [36]
**Nephrotic syndrome (NS)**	40	WT1	Differential UC	WB	-WT1 was detected in 25 patients (62.5%). -No significant correlation between WT1 amount and steroid responsiveness or renal pathological condition was found.	H. Lee, 2012 [37]
	13	miR-193a	-ExoQuick exosome precipitation (System Biosciences)	-qRT-PCR -ROC analysis -WB	-miR-193a was higher in children with primary FSGS than those in children with MCNS.	Z. Huang, 2017 [98]
	129 NS children and 126 age-/sex-matched HD.	miR-194-5p, miR-146b-5p, miR-378a-3p, miR-23b-3p and miR-30a-5p	UC	-High-throughput Illumina sequencing -qRT-PCR	-These 5 miRNAs were increased in NS and markedly reduced during the clinical remission period. -The concentrations of miR-194-5p and miR-23b-3p were positively correlated with the urine protein content and were markedly higher in the high urine protein group than in the low urine protein group.	T. Chen, 2019 [99]
**Glomerulonephritis (GN)**	12 IgAN, 12 TBMN patients and 6 HD	ANPEP, VASN, A1AT and CP	Differential UC	Label-free LC-MS/MS	-These four proteins are biomarkers to distinguish between early IgAN from TBMN.	P. G. Moon, 2011 [100]
	55 IgAN patients and 24 HD	CCL2 mRNA	Differential UC	qRT-PCR	- CCL2 was specifically expressed in uEVs of patients with GN compared to healthy controls. - Exosomal CCL2 was correlated with tubulointerstitial inflammation and C3 deposition in GN patients.	Y. Feng, 2018 [101]
**Lupus nephritis** **(LN)**	13 LN patients and 8 HD	miR-26a	Differential UC	qRT-PCR	-miR-26a levels in urinary exosomes were higher compared with healthy control.	O. Ichii, 2014 [46]
	41 SLE patients, 27 LN and 20 HD	miR-146a	UC	qRT-PCR	-Compared to controls, urinary level of miR-146a was higher in SLE patients.	J. Perez-Hernandez, 2020 [102]
	32 s patients with biopsy-proven LN, 15 non-lupus CKD and 20 HD	miR-29-c	Differential UC	qRT-PCR	-miR-29c correlated with the degree of renal chronicity but not with renal function. -MiR-29c expression levels could predict the degree of chronicity in patients with LN.	C. Solé, 2015 [103]
	31 patients	let-7a and miR-21	UC	qRT-PCR	-Urinary exosome-associated miRNA, let-7a and miR-21, could be used to guide the clinical stage of LN patients and possibly plays a role in epigenetic regulation of the kidney during the disease.	P. Tangtanatakul, 2019 [104]
**Ciliopathies**	12 ciliopathy patients and 12 age- and gender-matched HD	156 differentially expressed proteins	Differential UC	-Electro-phoresis -In-depth label-free LC-MS/MS proteomics analysis	-The most overexpressed or downregulated proteins in uEVs (VCAN, DPEP1 and FAT4) correlated with nephronophthisis-related ciliopathies.	M.F. Stokman, 2019 [105]
	1 ADPKD patient and multiple HD	PC1, PC2, TMEM2	-Differential UC - DG-UC	Gel electro-phoresis	In patients with PKD1 mutations, levels of PC1 and PC2 were reduced. -TMEM2 was higher in individuals with PKD1 mutations. -The PC1/TMEM2 ratio correlated inversely with height-adjusted total kidney volume in the discovery cohort. -The ratio of PC1/TMEM2 or PC2/TMEM2 could be used to distinguish individuals with PKD1 mutations from controls in a confirmation cohort.	M.C. Hogan, 2009 [106]
**Diabetic nephropathy (DN)**	48 type-1 diabetes mellitus patients and 25 HD	WT1	UC	WB	-WT1 expression in in uEVs was higher than in controls. -WT1 levels were associated with an increase in urine protein-to-creatinine ratio, albumin-to-creatinine ratio, and serum creatinine as well as a decline in eGFR.	A. Kalani, 2013 [107]
	30 HD and 30 T1D	miR-424 and miR-218	combined centrifugation	RT-qPCR	Association of urinary exosomal level of miR-424 and miR-218 with renal damage in T1D patients.	Q. Kong, 2019 [108]
	48	uEV miRNAs	qEV size exclusion columns (Izon Science)	NGS	Identification of a set of miRNAs with concentration changes associated with DN occurrence, microalbuminuria status, and other variables.	V. Ghai, 2018 [109]
**Obstructive nephropathy**	27 patients and 20 HD	AQP2 TGFβ-1 L1CAM	Centrifugation	Immuno-blotting	In boys with PUV, AQP2, TGFβ-1 and L1CAM correlated with eGFR.	P. Trnka, 2012 [110]
	3 UPJO and 3 healthy fetusis	633 differentially expressed proteins were identified in the amniotic fluid-derived exosomes from patients with UPJO.	Exosome Extraction Kit	iTRAQ quantitative proteomic profiles	- ACE and AP-N were significantly decreased in the amniotic fluid exosomes of women with a fetus diagnosed UPJO. - They correlate with suppressed cell proliferation, elevated ROS production, and increased pro-inflammatory cytokine levels in tubular cells.	R. Liu, 2020 [111]

HD: healthy controls; WT1: Wilm’s tumor protein-1; UC: ultracentrifugation; WB: western blot; FSGS: focal segmental glomerulosclerosis; SSNS: steroid-sensitive nephrotic syndrome; qRT-PCR: quantitative reverse transcription-polymerase chain reaction; ROC: receiver operating characteristic analysis; MCNS: minimal change nephrotic syndrome; ANPEP: aminopeptidase N; VASN: Vasorin precursor; A1AT: a-1-antitrypsin; CP: ceruloplasmin; LC-MS/MS: liquid chromatography mass spectrometry; IgAN: IgA nephropathy; TBMN: thin basement membrane nephropathy; SLE: systemic lupus erythematosus; CKD: chronic kidney disease; VCAN: versican; DPEP1: dipeptidase 1; FAT4: FAT Atypical cadherin 4; DG-UC: density-gradient centrifugation; TMEM2: transmembrane protein-2; ADPKD: Autosomal dominant polycystic kidney disease; PC1: polycystin-1; PC2: polycystin-2; eGFR: estimated glomerular filtration rate; T1D: type 1 diabetes; NGS: next generation sequencing; PUV: posterior urethral valves; AQP2: aquaporin-2; TGFβ1: transforming growth factor-1; L1CAM: L1 cell adhesion molecule; UPJO: Ureteropelvic junction obstruction; ACE: angiotensin-converting enzyme; and AP-N: aminopeptidase N.
